# Emergency Physicians as Good Samaritans: Survey of Frequency, Locations, Supplies and Medications

**DOI:** 10.5811/westjem.2015.11.28884

**Published:** 2016-01-12

**Authors:** Taylor W. Burkholder, Renee A. King

**Affiliations:** * Denver Health and Hospital Authority, Department of Emergency Medicine, Denver, Colorado; † University of Colorado School of Medicine, Department of Emergency Medicine, Aurora, Colorado

## Abstract

**Introduction:**

Little is known about the frequency and locations in which emergency physicians (EPs) are bystanders to an accident or emergency; equally uncertain is which contents of an “emergency kit” may be useful during such events. The aim of this study was to describe the frequency and locations of Good Samaritan acts by EPs and also determine which emergency kit supplies and medications were most commonly used by Good Samaritans.

**Methods:**

We conducted an electronic survey among a convenience sample of EPs in Colorado.

**Results:**

Respondents reported a median frequency of 2.0 Good Samaritan acts per five years of practice, with the most common locations being sports and entertainment events (25%), road traffic accidents (21%), and wilderness settings (19%). Of those who had acted as Good Samaritans, 86% reported that at least one supply would have been useful during the most recent event, and 66% reported at least one medication would have been useful. The most useful supplies were gloves (54%), dressings (34%), and a stethoscope (20%), while the most useful medications were oxygen (19%), intravenous fluids (17%), and epinephrine (14%).

**Conclusion:**

The majority of EPs can expect to provide Good Samaritan care during their careers and would be better prepared by carrying a kit with common supplies and medications where they are most likely to use them.

## INTRODUCTION

Off-duty emergency physicians (EPs) may be called upon to provide care as bystanders to accidents and emergencies outside of the hospital.^1^ The EP, with a unique skill set for rapid assessment and stabilization, is often the most qualified bystander to act as a “Good Samaritan,” named for the biblical parable of a Samaritan who aided an injured traveler on the roadside.^2^ Moreover, medical ethicists cite a moral imperative to provide such care when it is safe to do so.^3^,^4^

Anecdotes of EPs providing care outside of their official roles can be heard from the break room to popular emergency medicine podcasts. With the exception of Good Samaritan events on airplanes and in schools, little is known about how often or in which locations these events occur.^3^,^5^,^6^

Anticipating that most EPs will be bystanders to an emergency in their lifetime, several organizations worldwide have recommended carrying an emergency kit.^7^,^8^ Recommendations for kit contents are largely based on consensus or expert opinion, focusing on airway protection and hemorrhage control.^1^,^9^ An inventory of commonly used supplies and medications has yet to be established. This study aims to do the following:

1. Describe the (a) frequency and (b) locations of out-of-hospital emergencies in which EPs are called upon to provide Good Samaritan care.
2. Determine which supplies and medications are most frequently useful to EPs during Good Samaritan acts.

## METHODS

We emailed a link to an electronic survey to a convenience sample of board-certified and board-eligible EPs and pediatric EPs at five emergency departments along the Front Range of Colorado in April 2015. After obtaining written consent, the respondents were asked a total of eight multiple choice and fill-in-the-blank questions. Comments and anecdotes were welcomed at the end of the survey.

Survey responses were aggregated in Microsoft Excel (v.14.5.5, Redmond, WA) and analyzed with simple descriptive statistics. The survey was approved by the Colorado Multiple Institutional Review Board.

## RESULTS

**Figure 1:**
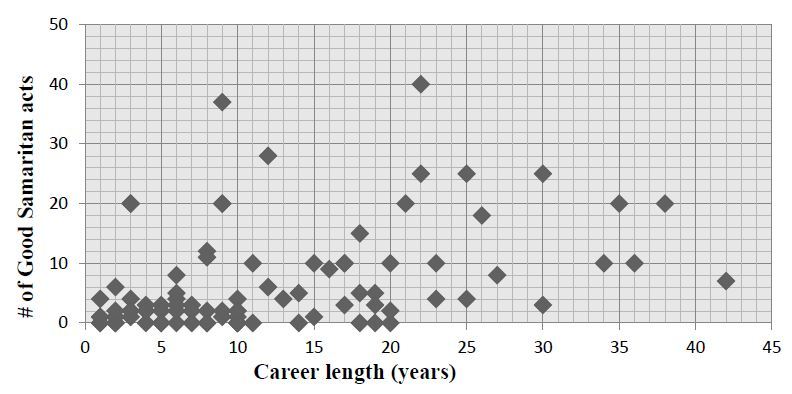
Reported career Good Samaritan acts.

A total of 90 responses were returned from the 167 invitations sent (response rate: 54%). During their careers as EPs, 78% (n=70) reported having provided Good Samaritan care. The median reported number of Good Samaritan acts was 2.0 events per five years in practice (IQR 0.5 to 4.2). Several outliers reporting very high numbers of Good Samaritan acts were noted (Figure 1).

**Figure 2:**
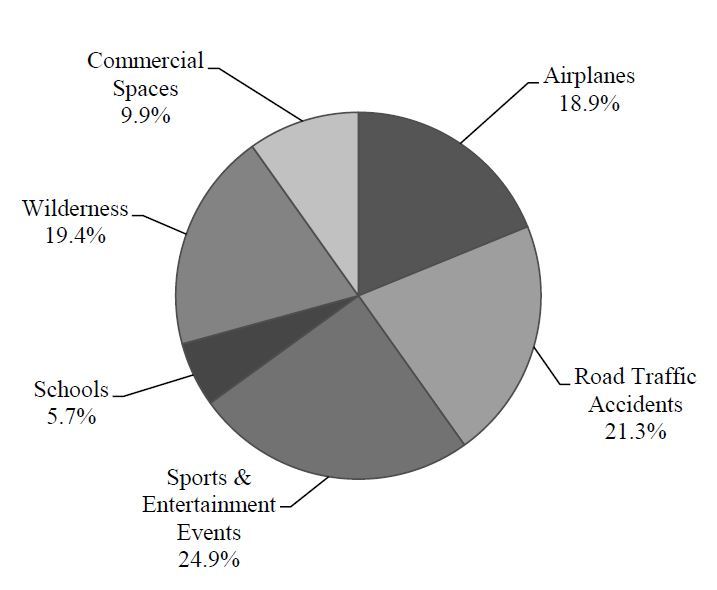
Locations of Good Samaritan events.

The locations of Good Samaritan events are reported in Figure 2. Sports and entertainment events were most common (24.9%, n=144), followed closely by road traffic accidents (21.3%, n=123), and wilderness settings (19.4%, n=109).

**Figure 3:**
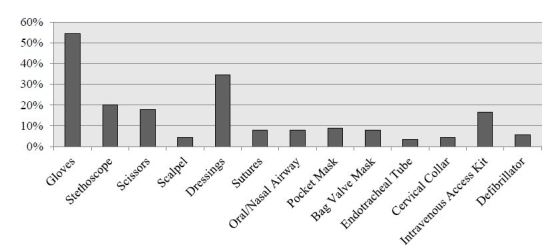
Supplies considered useful (by survey respondents).

**Figure 4:**
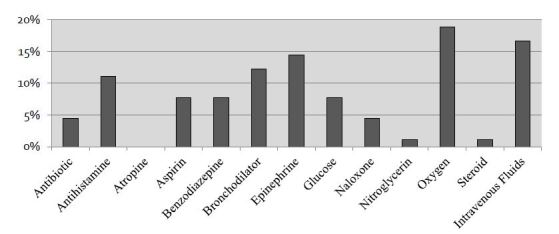
Medications considered useful (by survey respondents) in the event of Good Samaritan intervention.

Of the 70 respondents who reported previous experience as Good Samaritans, 86% (n=60) reported that at least one emergency kit supply would have been useful during their most recent Good Samaritan event, and 66% (n=46) reported that at least one medication would have been useful. The utility of supplies and medications is reported in Figures 3 and 4, respectively.

## DISCUSSION

The vast majority of surveyed EPs have performed Good Samaritan acts during their careers. Many of the respondents reported that at least one supply and one medication would have been useful. The high frequency of Good Samaritan acts and the reported utility of supplies and medications support the idea that EPs should be prepared in the event that they are bystanders to an accident or emergency.

One strategy to improve preparedness would be to carry a kit with commonly used supplies and medications while in the locations where Good Samaritan events most frequently occur. In this way, keeping an emergency kit in one’s car—which is often within reach of the EP at the scene of road traffic accidents or sports and entertainment events—would maximize access to useful supplies and medications in times of need.

## LIMITATIONS

This survey was limited by its retrospective nature. The reported frequency, locations, and utility of supplies and medications were all dependent on the EPs’ ability to recall events. Also, respondents eager to share their Good Samaritan experiences may have been more likely to complete the survey than those who did not have any experience. Validity outside of the state of Colorado would also need to be further assessed, especially regarding locations of Good Samaritan events.

In analyzing several of the extreme outliers, comments left at the end of the survey suggest that these respondents included events that were outside of the scope of Good Samaritan acts defined in the survey (e.g., suturing a laceration of a family member at home). This likely over-reports the median frequency of Good Samaritan acts.

Although commonly desired supplies and medications should influence the composition of an emergency kit, there are other factors that must be considered. Supplies geared toward airway protection (i.e., airway adjuncts, pocket masks) and bleeding control (i.e., tourniquets) may be infrequently used but have significant life-saving utility in certain circumstances. Cost, perishability, and environmental-specific factors should also influence emergency kit composition.

## CONCLUSION

The prepared emergency physician can expect to provide Good Samaritan care multiple times during a career. Many physicians report utility of supplies and medications during these events. Keeping this in mind, EPs may benefit from carrying a kit containing commonly used supplies and medications in situations where they are most likely to be called upon as Good Samaritans.
